# Interpersonal Problems and the Alternative Model of Personality Disorders: An Investigation Using the Interpersonal Circumplex

**DOI:** 10.1002/pmh.70045

**Published:** 2025-11-19

**Authors:** Peter L. A. Schiemainski, Julia I. Kunz, Sophie Nagl, Johannes Wolf, Stephan Goerigk, Andrea Jobst, Frank Padberg, Matthias A. Reinhard

**Affiliations:** ^1^ Department of Psychiatry and Psychotherapy LMU University Hospital Munich Germany; ^2^ Charlotte Fresenius Hochschule Munich Germany

**Keywords:** Alternative Model of Personality Disorders, interpersonal circumplex, interpersonal problems, personality functioning, personality traits

## Abstract

A central feature of personality disorders (PDs) is interpersonal problems, which can be effectively conceptualized using the interpersonal circumplex (IPC). This study replicates and extends previous research on the relationship between the dimensional DSM‐5 Alternative Model for Personality Disorders (AMPD) and interpersonal problems, as there are only a few studies in this area. The Structural Summary Method (SSM) was used in a sample of 168 psychiatric inpatients who completed Criterion A and B measures according to the AMPD: The Semi‐Structured Interview for Personality Functioning DSM‐5 (STiP‐5.1), the Level of Personality Functioning Scale (LPFS‐BF 2.0), and the Personality Inventory for DSM‐5 (PID‐5‐BF+). Additionally, general and specific interpersonal problems were assessed with the Inventory of Interpersonal Problems (IIP‐C). We found associations of both Criteria A and B of the AMPD with general interpersonal distress. Criterion A domains did not map cleanly onto the IPC's meta‐concepts of agency and communion; only a subset yielded interpretable circumplex profiles, primarily in the interpersonal functioning domain. Criterion B domains generally showed clearer and more specific associations, with most mapping onto domineering and cold interpersonal problems but showing little association with nonassertive or maladaptive warm problems. These results support and extend previous evidence of empirical links between the AMPD and the IPC, highlighting the IIP‐C's value for comprehensive assessment of interpersonal problems in PD. Further research is needed to clarify the overlap and distinctions between these models and to identify the interpersonal problems most relevant for treatment planning.

## 1 Introduction

Interpersonal problems are a key component of personality disorders (PDs) and encompass difficulties a person experiences in their relationships with others (Horowitz et al. 2016). These problems may arise from behaviors that are lacking (e.g., saying “no” to others) or excessive (e.g., arguing with others; Horowitz et al. 2016) and can be captured within the *interpersonal circumplex* (IPC; Fournier et al. 2011; Gurtman 1994).

**FIGURE 1:**
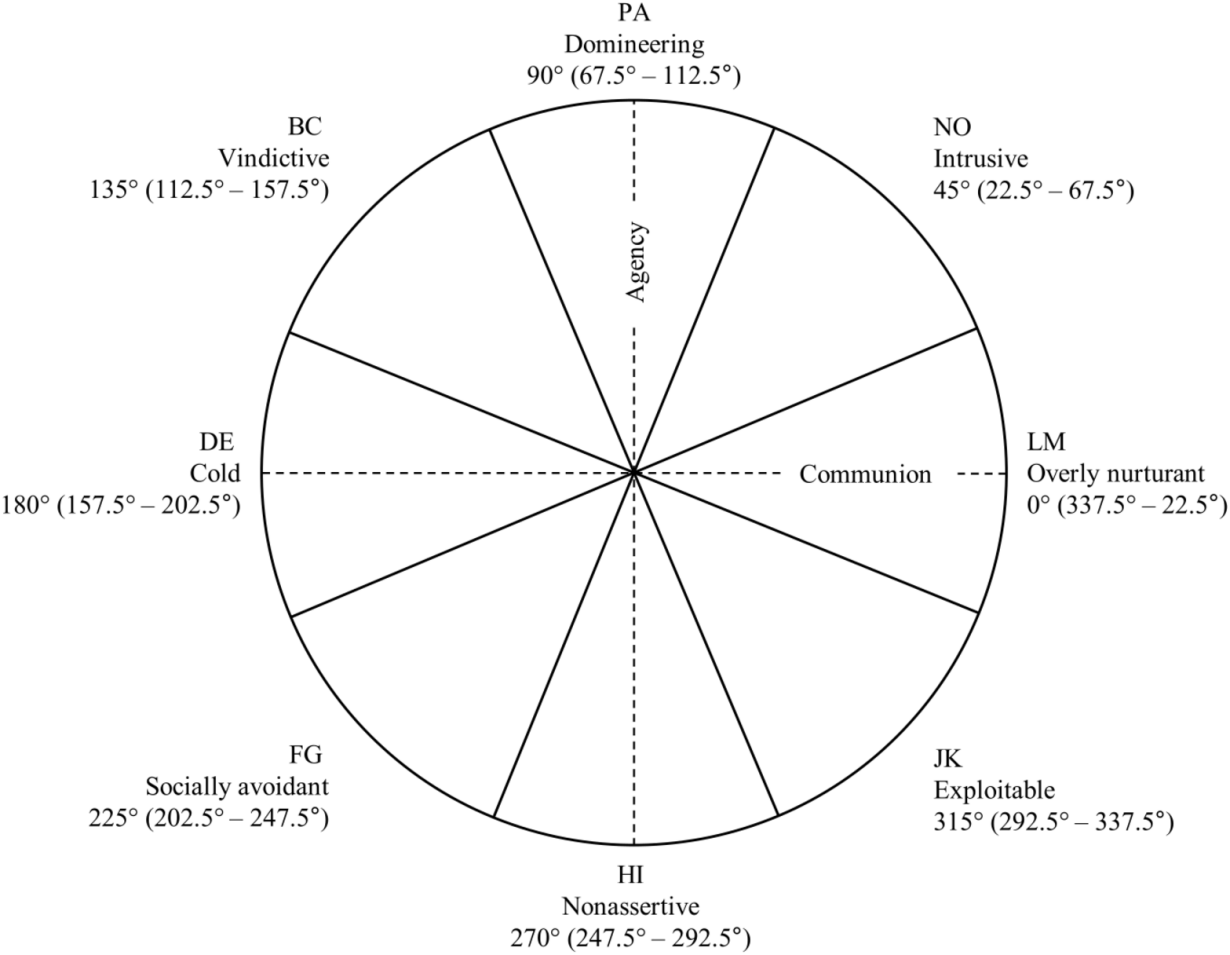
Interpersonal circumplex. *Note:* The dashed lines represent agency and communion; the solid lines define the octants, whose centers and spans are indicated by the degree numbers.

The IPC is a circular model based on structural assumptions, with two orthogonal bipolar axes: agency (vertical) and communion (horizontal). *Agency* reflects a person's sense of autonomy that manifests in pursuing power, mastery, and assertion (Fournier et al. 2011). Interpersonal problems within this concept range from domineering (i.e., maladaptive high agency) to nonassertive behavior (i.e., maladaptive low agency). *Communion* refers to being part of a larger social unit and manifests itself in the desire for intimacy, solidarity, and unity (Fournier et al. 2011). Interpersonal problems within this concept range from overly nurturant (i.e., maladaptive high communion) to cold behavior (i.e., maladaptive low communion). Every position on the IPC represents a blend of agency and communion, organized into eight equally sized sectors (“octants”), labeled PA through NO (Thomas and Strauß 2008; see Figure 1).

According to the Diagnostic and Statistical Manual of Mental Disorders, fifth edition (DSM‐5), PDs are defined as persistent, dysfunctional patterns in cognition, affectivity, and interpersonal behavior that are distressing, rigid, and stable over time (American Psychiatric Association 2013). Because of a variety of problems regarding the validity, reliability, and clinical utility of this categorical diagnostic system (e.g., Skodol 2012), and the consequent need for a more differentiated assessment of personality functioning (Hopwood et al. 2011), DSM‐5 introduced the Alternative Model for Personality Disorders (AMPD) as a dimensional diagnostic approach. A key innovation of the AMPD is the differentiation of moderate to severe impairments in personality functioning (Criterion A) as the foundation of all PDs, accompanied by problematic personality traits (Criterion B). Criterion A distinguishes self‐functioning (identity and self‐direction) and interpersonal functioning (empathy and intimacy). According to the AMPD, impairments of these basic adaptive capacities underlie all PDs and can be assessed using the Level of Personality Functioning Scale (LPFS). Criterion B captures 25 maladaptive traits to further characterize a PD, which are grouped into five domains: negative affectivity, detachment, antagonism, disinhibition, and psychoticism (American Psychiatric Association 2013). Prior research suggests that Criterion A and B measures may share substantial variance attributable to a broad general factor of personality pathology (Sharp et al. 2015; Wright et al. 2016). This factor is strongly associated with psychosocial functioning and may influence observed associations with other constructs, such as interpersonal problems.

The IPC's dimensions of agency and communion align conceptually with the “self” and “interpersonal” concepts of AMPD Criterion A. Agency maps onto self‐functioning (i.e., identity and self‐direction), with lower agency indicating impaired self‐image and self‐reflection, inconsistent goal‐directedness, and poor emotional regulation. Communion is linked to interpersonal functioning (i.e., empathy and intimacy), with lower communion corresponding to reduced empathy, reciprocity, and affiliation, focusing on self‐centered needs and harm avoidance (Pincus 2011).

Empirical research on this is still limited. For example, Dowgwillo et al. (2018) examined whether self‐reported impairments in personality functioning among 248 students could be mapped onto the IPC. They observed that impairments in interpersonal functioning were associated with low communion (angular displacement = 138°–174°, that is, octants BC–DE; please refer to the methods section for an explanation of the statistical model and parameters) and differentiated profiles (amplitude = 0.20–0.21; *R* ^2^ = 0.82–0.87). However, for impairments in self‐functioning, only self‐direction was linked to interpersonal problems of cold behavior (octant DE), aligning with low communion rather than maladaptive agency, though the profile was less differentiated (amplitude = 0.11, *R* ^2^ = 0.89). Identity showed no specific IPC pattern (*R* ^2^ = 0.57).

Within Criterion B, the domains of detachment (capturing the avoidance of socioemotional experiences) and antagonism (capturing conflictual interpersonal behavior) are considered to have the strongest interpersonal content because of their relational focus (American Psychiatric Association 2013). Detachment was found to be associated with interpersonal problems of being cold or socially avoidant (angular displacement = 201°–220°; i.e., octants DE–FG), with IPC profiles showing high prototypicality (*R* ^2^ = 0.81–0.90) and amplitude ranging from 0.12 to 0.29. Antagonism was associated with interpersonal problems of being domineering in student and clinical samples (angular displacement = 106°–107°; i.e., octant PA; Wright et al. 2012; Williams and Simms 2016; Liu et al. 2025). In Liu et al.'s offender sample, the full PID‐5 showed a shift toward octant BC (138.5°), whereas the short form stayed within octant PA (119.7°). Amplitude ranged from 0.09 to 0.28, with *R* ^2^ values between 0.75 and 0.95.

Although the other three domains capture more intrapsychic content, prior research found associations of negative affectivity with interpersonal problems of being overly nurturant or intrusive (octants LM–NO) with angular displacement ranging from 320° to 349° (Wright et al. 2012; Liu et al. 2025). An exception was found in Liu's offender sample, where the angular displacement shifted markedly to 180.5°. However, profile differentiation was limited overall: Only Wright et al. (2012) reported a differentiated profile (amplitude = 0.16), whereas Liu et al. (2025) found very low amplitude (0.04–0.05), suggesting weak interpersonal specificity. Disinhibition has been associated with domineering or vindictive behavior (octants PA–BC), with angular displacement between 114° and 129°. Wright et al. (2012) reported a differentiated profile (amplitude = 0.16, *R* ^2^ = 0.92), whereas Williams and Simms (2016) and Liu et al. (2025) found less differentiated profiles (amplitude = 0.04–0.07, *R* ^2^ = 0.73–76). For psychoticism, prior research found less consistent interpersonal profiles, typically located near the vindictive region (angular displacement = 120°–147°; i.e., octant BC; *R* ^2^ = 0.90–0.93; Williams and Simms 2016; Wright et al. 2012), but amplitudes remained low (0.10–0.12). In Liu's offender sample, the angular displacement shifted to 205.1°, suggesting a socially avoidant style (octant FG), but the profile remained weakly differentiated (*R* ^2^ = 0.83, amplitude = 0.06).

Given the limited evidence for the overlap between AMPD and IPC, this study aimed to examine interpersonal problem profiles of criteria A and B using the IPC as a nomological framework. In doing so, we build on prior work that applied the same SSM to AMPD constructs in student and clinical samples, allowing for a systematic replication and extension of earlier findings. In contrast to previous work, this is the first study to do so in a mixed psychiatric sample with high PD prevalence, using both Criteria A and B. In addition, we applied not only self‐rated assessments, but also a clinician‐rated interview of Criterion A to investigate the generalizability of our findings.

We hypothesized that

Hypothesis 1 *AMPD Criterion A impairments would relate to general interpersonal distress*.

Furthermore, we assumed that

Hypothesis 2 *Impairments in interpersonal functioning would be associated with interpersonal problems reflecting low communion (i.e., vindictive, cold, and socially avoidant)*.

Because of inconsistent findings (Dowgwillo et al. 2018), no hypothesis was made for impairments in self‐functioning.

Hypothesis 3 *Regarding Criterion B, all five domains were expected to be linked to general interpersonal distress*.

Following previous findings (Williams and Simms 2016; Wright et al. 2012), we assumed that detachment would be most strongly associated with social avoidance (octant FG), antagonism with domineering behavior (octant PA), negative affectivity with being overly nurturant (octant LM) or intrusive (octant NO), disinhibition with vindictive (octant BC) or domineering behavior (octant PA), and psychoticism with vindictiveness (octant BC).

## 2 Method

### 2.1 Participants and Procedure

Data were collected at the Department of Psychiatry and Psychotherapy, LMU University Hospital, Munich, Germany, as part of a naturalistic study (German Clinical Trial Register ID: DRKS00019821), approved by the local ethics committee (EK‐No. 713–15). Participants were inpatients, aged 18–65, enrolled in one of two 10‐week psychotherapy programs, primarily for borderline PD (BPD) or persistent depressive disorder. Exclusion criteria included acute suicidality, unstable somatic conditions (e.g., acute and chronic infections), and pregnancy.

All participants gave written informed consent before completing self‐report measures and diagnostic interviews, including the SCID‐5‐CV and SCID‐5‐PD (Beesdo‐Baum et al. 2019a, 2019b). Interviews were conducted and evaluated by trained psychiatrists, clinical psychologists, or supervised advanced psychology students. All assessments were administered within the first week after intake to ensure comparability.

Data from *N* = 168 patients (63.7% female, 3.0% gender diverse; *M*_age_ = 34.01 years, SD = 13.05) were included. Of these, 11 (6.5%) did not complete the Inventory of Interpersonal Problems (IIP‐C), 4 (2.4%) did not complete the LPFS‐BF 2.0, 6 (3.6%) did not complete the PID‐5‐BF+, and 28 (16.7%) did not complete the STiP‐5.1, primarily due to treatment discontinuation.

A total of 154 patients completed the SCID‐5‐CV and SCID‐5‐PD, with 124 (80.5%) meeting criteria for major depressive disorder, 112 (72.7%) for persistent depressive disorder, 88 (57.1%) for at least one anxiety disorder, 12 (7.8%) for obsessive–compulsive disorder, 66 (42.9%) for PTSD, and 33 (21.4%) for substance abuse (mainly alcohol or cannabis).

Of 78 patients diagnosed with at least one categorical personality disorder, 54 (35.1%) had one PD, 16 (10.4%) had two, and 8 (5.2%) had three categorical PDs. Of these 110 diagnoses, 54 (49.1%) were BPD, 35 (31.8%) avoidant PD, and 14 (12.7%) obsessive–compulsive PD. Paranoid PD and dependent PD were each diagnosed twice (1.8% each), schizoid PD and antisocial PD once (0.9% each). A total of 76 patients (49.4%) did not meet the criteria for any PD.

### 2.2 Measures

#### 2.2.1 Alternative Model for Personality Disorders

Criterion A personality functioning was assessed via interview and self‐report. Clinician ratings were obtained using the German Semi‐Structured Interview for Personality Functioning DSM‐5 (STiP‐5.1; Hutsebaut et al. 2017), which is based on the LPFS consisting of 12 sections with 28 open questions, followed by additional questions if further clarification is required (Zettl et al. 2019). Impairment is rated from 0 (little or none) to 4 (extreme). Overall impairment can be differentiated into two domain scores, each with two subdomains (self‐functioning: identity, self‐direction; interpersonal functioning: empathy, intimacy; Hutsebaut et al. 2017). The German STiP‐5.1 showed high internal consistency (*α* = 0.96) and high interrater reliability for the facets (ICC = 0.79–0.92) and the total score (ICC = 0.93; Zettl et al. 2019).

Self‐reported personality functioning was assessed using the Level of Personality Functioning Scale‐Brief Form 2.0 (LPFS‐BF 2.0; Weekers et al. 2019). It contains one item for each of the 12 facets of the LPFS and therefore six items each for self‐ and interpersonal functioning. Items are rated from 1 (very false or often false) to 4 (very true or often true). The total score and both domain scores reflect the average of completed items, with higher scores indicating greater impairment. The German LPFS‐BF 2.0 showed high reliability (*ω*_total_ > 0.9; *ω*_domains_ > 0.8; Spitzer et al. 2021) and good convergent validity (Spitzer et al. 2021; Weekers et al. 2019). In this study, the LPFS‐BF 2.0 showed good reliability for the total score (*ω* = 0.77) and subscales (*ω* = 0.70–0.73).

Criterion B of the AMPD, that is, problematic personality traits, was assessed with the Personality Inventory for DSM‐5, Brief Form Plus (PID‐5‐BF+; Kerber et al. 2022), a 34‐item short form of the Personality Inventory for DSM‐5 (PID‐5; Krueger 2013). Items are rated from 0 (very false or often false) to 3 (very true or often true). Scores are calculated as averages for total, domain, and facet levels, with higher scores indicating more pronounced problematic personality traits. The German PID‐5‐BF+ was validated in two non‐clinical samples (*N* = 1342; Kerber et al. 2022) and internal consistency was examined in a population‐representative sample (*N* = 4727), showing values between *ω* = 0.77 and *ω* = 0.86 for the five domains (Rek et al. 2021). In this study, the PID‐5‐BF+ demonstrated good total reliability (*ω* = 0.87) with subscale values between *ω* = 0.69 and 0.80.

#### 2.2.2 Interpersonal Problems

Interpersonal problems were assessed with the German version of the IIP‐C (German version: IIP‐D, 3rd ed.; Horowitz et al. 2016). The IIP‐C includes eight subscales (one per IPC octant), each capturing a specific problematic interpersonal behavior with eight items rated from 0 (not at all) to 4 (extremely). Thus, IIP‐C operationalizes interpersonal problems as a lack or excess of behaviors on the IPC axes of agency and communion. Factor structure was confirmed in three samples. Total score and scores for the eight subscales are computed as means, with higher scores indicating more pronounced interpersonal problems. Internal consistency ranged from *α* = 0.71 to *α* = 0.82 (Horowitz et al. 2016). In this sample, the IIP‐C exhibited excellent total reliability (*ω* = 0.90) and subscale reliabilities ranging from *ω* = 0.73 to 0.86.

### 2.3 Statistical Analysis

Statistical analyses were conducted using IBM SPSS Statistics (version 29) and R (version 4.3.2), with a significance threshold of *α* = 0.05.

Due to the complex structure of the IIP‐C data, linear correlations with the AMPD data were insufficient to capture the geometric assumptions of the IPC, where neighboring scales (e.g., domineering and intrusive) are conceptually similar and positively correlated, orthogonal scales are unrelated and correlate near zero (e.g., domineering and cold), and opposite scales are negatively correlated (i.e., overly nurturant and cold as the two ends of the communion spectrum; Gurtman and Pincus 2003).

To address this, the Structural Summary Method (SSM; e.g., Gurtman and Balakrishnan 1998; Wright et al. 2009; Zimmermann and Wright 2017) was employed, modeling a cosine curve from correlations between IIP‐C scales and external measures (in this study, the scales of STiP‐5.1, LPFS‐BF 2.0, PID‐5‐BF+) to determine their placement in the IPC's nomological network, accounting for IIP‐C scale intercorrelations. The more the correlation pattern resembles an ideal cosine curve, the better the external measure reflects interpersonal problems (Zimmermann and Wright 2017).

**FIGURE 2:**
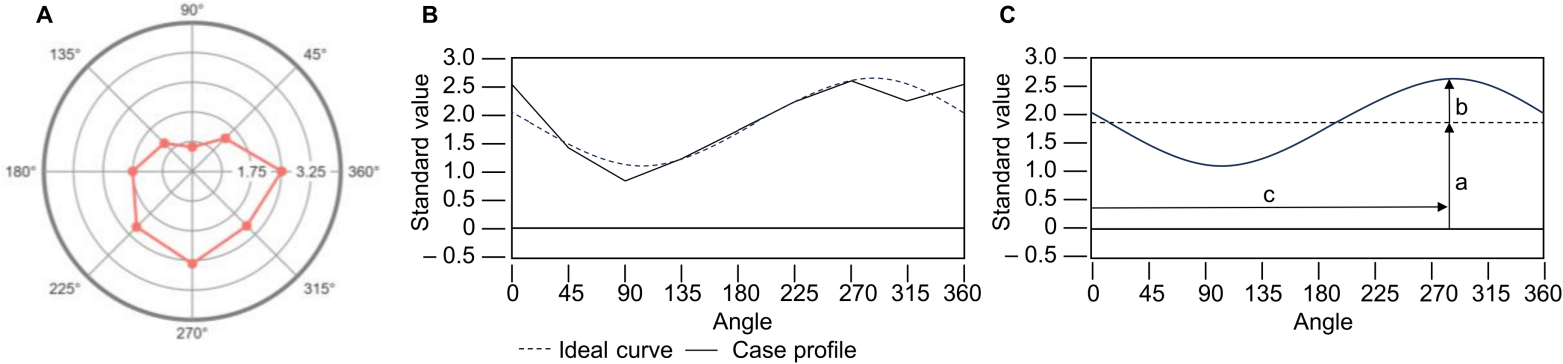
Graphical representation of the steps of the Structural Summary Method. *Note:* Figure 2A was generated with R.

Figure 2 illustrates the process: The eight IIP‐C scales are mapped in the IPC space (2A), a curve is derived (2B), and finally the comparison of this curve with the ideal cosine curve is summarized with the structural parameters elevation (a), amplitude (b) and angular displacement (c) (2C; Thomas and Strauß 2008). If the model fit (*R* ^2^, not shown in Figure 2) of an IIP‐C profile is sufficiently high (≥ 0.80), it can serve as a representation of the IPC and provide a reliable basis for comparison with external measures (Wright et al. 2009). In this case, *elevation* reflects general interpersonal distress as the uniform correlation of an external measure across all IIP‐C scales. *Amplitude* indicates profile differentiation by showing the extent to which an external measure correlates with the scales of the IIP‐C to different degrees and specifically (Vittengl et al. 2022) with larger values indicating that a scale relates to a specific interpersonal problem in the IIP‐C. The parameter *R* ^2^ indicates how well the observed correlation pattern of a scale matches the prototypical cosine curve. If an external measure has a prototypical correlation profile (*R* ^2^ > 0.7; Wright et al. 2009), its amplitude can be interpreted. In addition, if the amplitude is marked (i.e., the scale shows specific relationships with the IIP‐C), the *angular displacement* can be interpreted, indicating the octant of IPC to which the scale is most strongly related (i.e., the type of interpersonal problems).

For the present study, the established heuristic threshold value of ≥ |0.15|was used to identify markedly differentiated or elevated profiles (Wright et al. 2012; Zimmermann and Wright 2017). Fisher's *r*‐to‐*z* transformation (Steiger 1980) was applied to compare the associations of self‐reported and clinician‐observed impairments in personality functioning with the overall level of interpersonal difficulties as measured by the IIP‐C total score.

Additionally, 95% confidence intervals (CIs) around SSM parameters were calculated using bootstrapping (5000 iterations; Zimmermann and Wright 2017). Effect sizes were interpreted with thresholds of |*d*| = 0.20 (small), 0.50 (medium), and 0.80 (large), and correlation coefficients were considered small from |*r*| = 0.10, medium from |*r*| = 0.30, and large from |*r*| = 0.50 (Cohen 2013).

## 3 Results

### 3.1 Sample

Data from *N* = 168 patients were available, with missing data occurring only at the instrument level, rather than only individual items within a measure. No significant differences in age or gender were found for STiP‐5.1, LPFS‐BF 2.0, or PID‐5‐BF+. Participants missing IIP‐C data were significantly younger (*p* = 0.037). Absence of the STiP‐5.1 interview was not associated with systematic differences in self‐report scores on LPFS‐BF 2.0, PID‐5‐BF+, or IIP‐C (all *p* > 0.38). Patients with at least one categorical PD showed significantly higher levels of overall interpersonal difficulties (IIP‐C total score: *t*(138.88) = 5.41, *p* < 0.001, *d* = 0.90), greater impairment in total personality functioning (STiP‐5.1: *t*(131.40) = 6.98, *p* < 0.001, *d* = 1.21; LPFS‐BF 2.0: *t*(146.37) = 7.18, *p* < 0.001, *d* = 1.18), and more problematic personality traits (PID‐5‐BF+ total score: *t*(146.96) = 7.21, *p* < 0.001, *d* = 1.18) than those without a categorical PD diagnosis.

The SSM revealed a strong circumplex structure for the IIP‐C in this sample, with an overall model fit of *R* ^2^ = 0.859. These results justify subsequent analyses interpreting AMPD measures within the circumplex framework.

### 3.2 AMPD Criterion A and IIP‐C

Supporting Hypothesis 1, both clinician‐observed and self‐reported impairments in personality functioning were associated with increased general interpersonal distress, as shown by elevation parameters for STiP‐5.1 (total: 0.26, self: 0.24, and interpersonal: 0.23) and LPFS‐BF 2.0 (total: 0.45, self: 0.38, and interpersonal: 0.37). Table 1 displays structural summary parameters and 95% CIs for STiP‐5.1, LPFS‐BF 2.0, and PID‐5‐BF+.

**TABLE 1:** Structural parameters with 95% CI for the AMPD scales in the IIP‐C space.

Scale	Elevation	Amplitude	Angular displacement	*R* ^2^
STiP‐5.1 total score	0.26 [0.16, 0.35]	0.07 [0.02, 0.20]	155.8 [28.7, 281.0]	0.60
Self‐functioning	0.24 [0.14, 0.33]	0.08 [0.02, 0.20]	233.5 [122.3, 333.8]	0.65
Identity	0.23 [0.14, 0.32]	0.06 [0.02, 0.19]	319.8 [189.9, 102.0]	0.40
Self‐direction	0.19 [0.08, 0.28]	0.14 [0.05, 0.25]	212.9 [158.4, 267.8]	0.89
Interpersonal functioning	0.23 [0.13, 0.32]	0.14 [0.05, 0.26]	116.9 [64.8, 170.6]	0.93
Empathy	0.17 [0.06, 0.26]	0.23 [0.13, 0.34]	99.8 [71.2, 129.1]	0.98
Intimacy	0.22 [0.12, 0.32]	0.08 [0.02, 0.22]	168.5 [43.7, 289.8]	0.68
LPFS‐BF 2.0 total score	45 [0.35, 0.53]	0.09 [0.02, 0.19]	121.9 [63.0, 206.9]	0.82
Self‐functioning	0.38 [0.29, 0.47]	0.03 [0.01, 0.15]	319.5 [157.0, 112.0]	0.25
Interpersonal functioning	0.37 [0.28, 0.46]	0.18 [0.09, 0.29]	124.8 [98.1, 156.8]	0.98
PID‐5‐BF+ total score	0.41 [0.33, 0.49]	0.16 [0.07, 0.26]	89.9 [53.3, 119.4]	0.91
Negative affectivity	0.34 [0.25, 0.41]	0.18 [0.10, 0.28]	40.1 [8.0, 70.6]	0.82
Detachment	0.30 [0.22, 0.38]	0.29 [0.22, 0.38]	214.5 [191.0, 234.3]	0.81
Antagonism	0.14 [0.03, 0.24]	0.27 [0.18, 0.37]	87.8 [63.6, 105.5]	0.94
Disinhibition	0.29 [0.20, 0.38]	0.17 [0.08, 0.26]	79.2 [46.8, 106.0]	0.99
Psychoticism	0.23 [0.13, 0.32]	0.17 [0.07, 0.29]	88.6 [52.6, 116.1]	0.93

*Note:* Lowest pair‐wise *N* by measure: STiP‐5.1, *N* = 128; LPFS‐BF 2.0, *N* = 153; and PID‐5‐BF+, *N* = 151. *Elevation* is an indicator of general interpersonal distress. *Amplitude* characterizes the degree of differentiation of the interpersonal profile. *Angular displacement* indicates the octant of IPC with the strongest relation (i.e., the type of interpersonal problems; see Figure 1). *R* ^2^ indicates the extent to which the observed correlation pattern of a scale matches the prototypical cosine curve.

**FIGURE 3:**
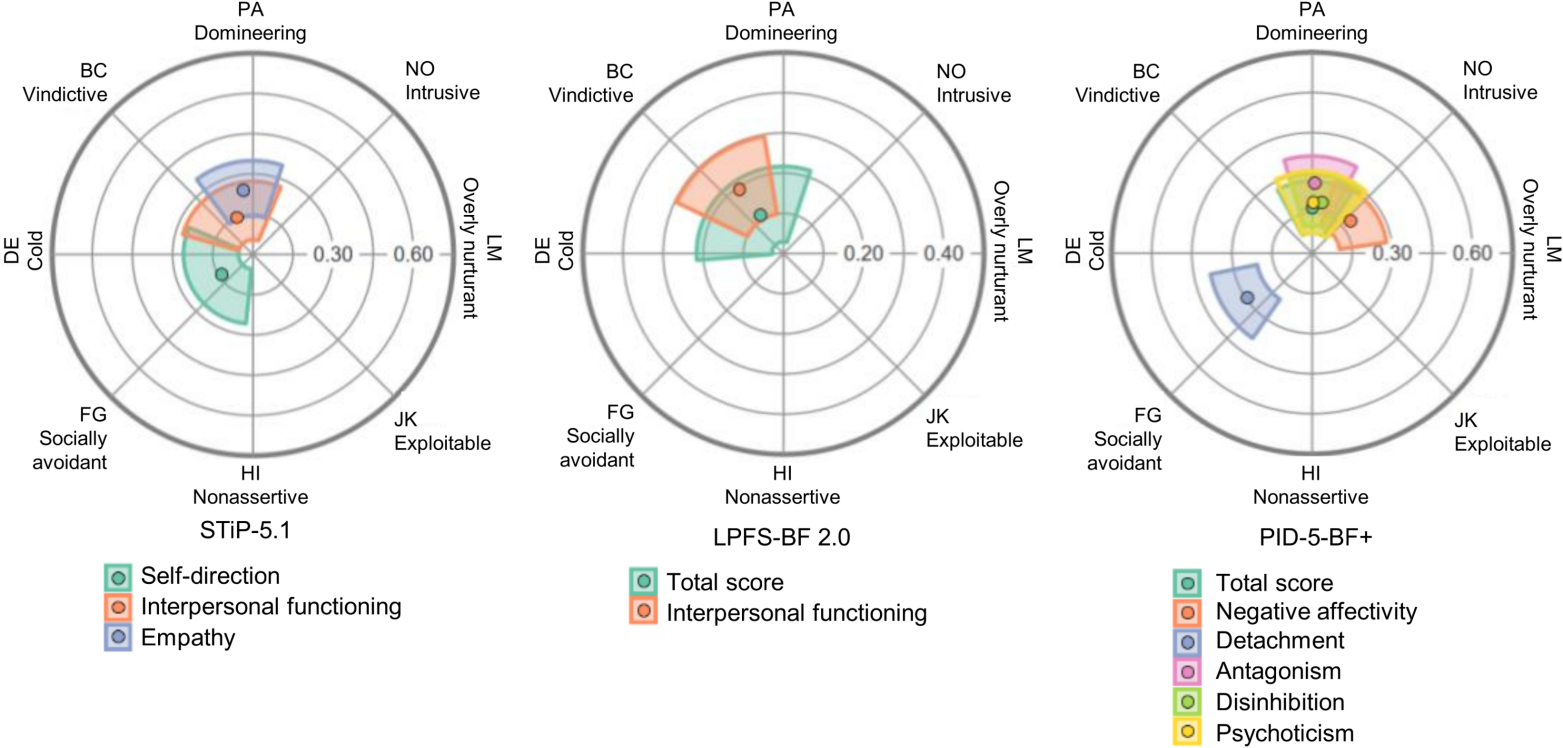
Projection of STiP‐5.1, LPFS‐BF 2.0, and PID‐5‐BF+ scales into the IIP‐C space. *Note:* Point estimates and 95% CIs are shown, resulting from amplitude (radial) and angular displacement (angular) of the respective scale. 0° is located at LM, from which angular displacement proceeds counterclockwise. Only domains with at least acceptable *R* ^2^ are shown; elevation is not included in the figures.

In addition, both interpersonal functioning scales of STiP‐5.1 and LPFS‐BF 2.0 showed acceptable *R* ^2^ values. Both scales were associated with vindictive interpersonal problems (octant BC), which, although not perfectly aligned with communion, fell within the expected range and thus supported hypothesis 2. Figure 3 depicts the placement of all scales with acceptable *R* ^2^ in the IPC. The STiP‐5.1 association, however, must be viewed with caution because its amplitude fell below the threshold (Table 1). Analysis of STiP‐5.1 subscales showed that intimacy lacked acceptable *R* ^2^, whereas empathy was associated with domineering interpersonal problems (octant PA) and therefore almost exclusively with maladaptive agency. Among self‐functioning, only the STiP‐5.1 subdomain self‐direction could be analyzed; it correlated with both low communion and low agency but with low amplitude, warranting cautious interpretation. To address potential interest, we provide zero‐order correlations between untransformed IIP‐C octant scores and AMPD Criterion A and B variables in the supplement (Table S1).

Self‐reported impairments in personality functioning were significantly and more strongly associated with overall interpersonal difficulties as measured by the IIP‐C total score than clinician‐rated impairments, as Fisher's *r*‐to‐*z* transformation showed (overall value: *z* = −3.70, *p* < 0.001; self‐functioning: *z* = −2.37, *p* = 0.018; and interpersonal functioning: *z* = −2.41, *p* = 0.016).

Self‐functioning and interpersonal functioning also correlated significantly within both measures (LPFS‐BF 2.0: *r* = 0.39, *p* < 0.001; STiP‐5.1: *r* = 0.61, *p* < 0.001). Moderate correlations were found between self‐reported and clinician‐observed total scores of LPFS‐BF 2.0 and STiP‐5.1 (*r* = 0.45, *p* < 0.001) as well as between their domains self‐functioning (*r* = 0.43, *p* < 0.001) and interpersonal functioning (*r* = 0.46, *p* < 0.001).

### 3.3 AMPD Criterion B and IIP‐C

As hypothesized (Hypothesis 3), the overall PID‐5‐BF+ score (elevation: 0.41) and four of its five domains (elevation: 0.23–0.34, except antagonism at 0.14) were associated with increased general interpersonal distress. All PID‐5‐BF+ scales showed *R* ^2^ > 0.80. Detachment and antagonism exhibited the largest amplitudes, followed by negative affectivity, disinhibition, and psychoticism.

As hypothesized, detachment was associated with socially avoidant interpersonal problems (octant FG), antagonism with domineering behavior (octant PA), negative affectivity with intrusiveness (octant NO), and disinhibition with domineering behavior (PA). Psychoticism was also—contrary to expectations—associated with domineering problems (PA) rather than vindictiveness (BC).

## 4 Discussion

Our study demonstrated a substantial overlap between the AMPD and the IPC. Both self‐reported and clinician‐rated impairments in overall functioning, self‐functioning, and interpersonal functioning were associated with general interpersonal distress and with specific interpersonal problems. The SSM proved particularly useful by differentiating between the severity of general interpersonal distress (elevation) and the type of interpersonal problems (amplitude, angular displacement) while preserving the circumplex structure. Although the latter can be described as reflecting interpersonal “style” (e.g., Vittengl et al. 2022), it is important to note that the IIP‐C specifically assesses interpersonal problems (Horowitz et al. 2016). We therefore interpret amplitude and angular displacement as clinically meaningful indicators of problem styles, that is, the characteristic ways in which interpersonal problems manifest—even when general interpersonal distress is relatively low, for example, when a single octant scale is pronounced while others remain low.

Our findings replicate and extend those of Dowgwillo et al. (2018), who showed that Criterion A domains do not map cleanly onto the IPC meta‐concepts of agency and communion. We observed similar patterns in a clinical inpatient sample characterized by a high rate of PD and therefore interpersonal problems and extend this finding by using not only self‐report measures but also a clinician‐rated measure of personality functioning. Interestingly, empathy and self‐direction in our study were linked to blends of maladaptive agency and low communion, supporting the view that Criterion A reflects complex interpersonal profiles. However, only four of the 10 Criterion A variables showed circumplex parameters that were clearly interpretable based on amplitude and prototypicality criteria, three of which were interpersonal functioning variables. Most self‐functioning domains and both total scores did not yield interpretable profiles. This limits the strength of conclusions that can be drawn about how Criterion A domains map onto specific interpersonal problem profiles. A key difference concerns the intimacy domain: While Dowgwillo et al. (2018) found a strong circumplex fit (*R* ^2^ = 0.87), the intimacy scale in our data fell below the threshold (*R* ^2^ = 0.68), suggesting that the psychometric properties of Criterion A measures may vary across instruments or populations.

Concerning Criterion B, the PID‐5‐BF+ total score and four of its five domains (except antagonism) were associated with elevated general interpersonal distress. All scale correlation patterns fitted an ideal cosine profile (*R* ^2^ > 0.80). The total score was mainly associated with domineering problems (octant PA), thus with maladaptive agency.

Negative affectivity, defined in DSM‐5 as frequent and intense experiences of high levels of negative emotions and their behavioral and interpersonal manifestations, was associated with intrusive interpersonal problems (octant NO) and not with overly nurturant behavior (octant LM). Regarding content, octant NO fits better with the negative affectivity domain, as it captures problems of people who place too much value on attention from others, feel responsibility for others, or have difficulties being alone.

In line with previous findings (Williams and Simms 2016; Wright et al. 2012), detachment was associated with the socially avoidant interpersonal problem type (octant FG). According to DSM‐5, detachment can be defined as avoidance of socioemotional experiences, focusing on withdrawal from interpersonal interactions, and the limited experience and expression of feelings. In line with this, people with high scores on the FG scale of the IIP‐C report problems in socializing, approaching others, and expressing their feelings.

As expected, antagonism was associated with the octant PA and thus with domineering interpersonal problems. Antagonism is defined in the DSM‐5 as behaviors that bring a person into conflict with others, including an exaggerated sense of self‐importance, as well as a callous antipathy towards others. In line with this, people with high scores on the PA scale in the IIP‐C report problems accepting others, wanting to change or influence them too much, being overly controlling, or frequently in conflict with others.

Disinhibition was also associated with domineering interpersonal problems (octant PA). This domain is defined in DSM‐5 as focusing on immediate gratification, which leads to impulsive behavior without regard for consequences and, therefore, may not perfectly match the content of the two octants PA and BC. However, the observed association with maladaptive agency in general seems plausible.

The domain of psychoticism, defined in the DSM‐5 as exhibiting a wide range of culturally incongruent, odd, eccentric, or unusual behaviors and cognitions, was also associated with octant PA, and not as hypothesized with vindictive interpersonal problems (octant BC), for which there were at least indications in other studies (Williams and Simms 2016; Wright et al. 2012). However, despite our findings, the octant BC may theoretically overlap better with the domain of psychoticism, because people with a predominant interpersonal profile located in this octant report problems with trusting others or being too suspicious, arguing too much, and being too revengeful, which may be directly or indirectly associated with psychoticism as described by AMPD.

Summarizing our findings regarding Criterion B, detachment and antagonism fell into the expected IPC octant FG and PA, respectively. In contrast, psychoticism, disinhibition, and negative affectivity were associated with octants reflecting higher levels of maladaptive agency than expected (i.e., PA and NO instead of the expected neighboring octants BC and LM). This stronger association with maladaptive agency could be due to different scoring methods for these three domains. Although Wright et al. (2012) used all 25 facets of the PID‐5 for domain scoring, Williams and Simms (2016) mapped the domains with only three facets each, as in the present study.

The results of the present study replicate and extend earlier findings showing that maladaptive warm and submissive interpersonal difficulties are insufficiently captured by the PID‐5. This limitation was first demonstrated by Wright et al. (2012) and has recently been reaffirmed by Ringwald et al. (2023), both using the full version of the PID‐5. Our findings indicate that this limitation also holds for the brief form (PID‐5‐BF+) and further extends to current measures of Criterion A, namely, the LPFS‐BF 2.0 and STiP‐5.1.

In this respect, the PID‐5 may show some content‐related limitations like other models for maladaptive personality traits (Hopwood et al. 2013, 2009; Vittengl et al. 2022). In the categorical PD model, these aspects were partly captured by measures of histrionic and dependent PD (Bornstein 2011). However, warm and submissive interpersonal behavior may not generally lead to interpersonal problems and, consequently, may be less clinically relevant in the context of PD diagnoses (Hopwood et al. 2013). It is also possible that maladaptive warm behaviors are less strongly perceived or reported by patients (Williams and Simms 2016), although they are present and lead to interpersonal problems, as Williams et al. (2014) suggest. Adaptive personality traits may better assess (maladaptive) interpersonal warmth and submissiveness (Williams and Simms 2016). For example, Liu et al. (2025) suggest that traits of the Big Five model capture maladaptive warm and submissive interpersonal problems. Items from other measures could therefore complement the AMPD to include these aspects (Gore and Widiger 2015; Simms et al. 2011).

Finally, our findings may inform clinical practice. The SSM parameters allow differentiation between general interpersonal distress (elevation) and their characteristic configuration (amplitude and angular displacement). This differentiation helps clinicians identify maladaptive problem styles (e.g., domineering/vindictive vs. socially avoidant/nonassertive) and tailor interventions accordingly, for instance, by focusing on impulse control and perspective taking versus self‐assertion and boundary setting. Moreover, awareness of patients' interpersonal styles can prepare therapists for alliance challenges (e.g., mistrust in vindictive styles and withdrawal in avoidant styles) and may help prevent alliance ruptures or premature dropout.

## 5 Limitations

The naturalistic, cross‐sectional design of this clinical study leads to some limitations. First, causal inference is limited, because only admission data were used, and personality functioning, problematic personality traits, and interpersonal problems, although relatively stable, reflect dynamic processes that warrant a longitudinal study design (Roche et al. 2016). Second, focusing solely on interpersonal problems may overlook the complexity of interpersonal processes and the impact of concurrent psychopathology, such as depressive or anxious symptoms. Third, despite the good external validity of our heterogeneous sample, BPD and avoidant PD dominated categorical diagnoses, and some PD types were absent, limiting generalizability. Fourth, self‐report measures may be biased by social desirability (Martí Valls et al. 2023) or difficulties in self‐reflection common in PDs (Carnovale et al. 2019). Additionally, interrater reliability was not assessed for diagnostic interviews, which may limit the consistency of their results. Fifth, as in other studies, some scales showed *R* ^2^ values below 0.70, suggesting multiple underlying problem patterns rather than a single clearly definable construct, which may reflect measurement error or sample‐specific characteristics (Gurtman and Balakrishnan 1998; Haslam and Gurtman 1999). Finally, Criterion B traits were assessed only by self‐report using the PID‐5‐BF+, a shortened PID‐5 which does not cover all facets of Criterion B and omits interpersonally relevant aspects such as maladaptive warmth, which may also be relevant for PDs (Krueger and Markon 2014).

Beyond methodological constraints, some limitations pertain to the findings themselves. For Criterion A, only four of 10 variables yielded interpretable profiles (see Table 1), limiting conclusions about the alignment between Criterion A domains and specific interpersonal problems. By contrast, all six Criterion B variables met amplitude and angular displacement thresholds, enabling a more robust interpretation of their circumplex placement. Moreover, although our analyses focused on domain and total scores, previous research indicates that these scales may share variance attributable to a general factor of personality pathology (Sharp et al. 2015; Wright et al. 2016). Such a factor could partially account for the strong associations between IIP‐C elevation and AMPD total scores, as well as certain unexpected findings. Future work could disentangle general and domain‐specific contributions, for instance, via bifactor modeling. Additional research is also warranted to clarify the clinical relevance of interpersonal problems insufficiently captured by Criterion B, ideally including external assessments of both personality traits and interpersonal problems.

Despite these limitations, our study has notable strengths. A key strength is the multidimensional assessment of Criterion A using both a semistructured clinician interview and a self‐report instrument. Interestingly, self‐reported overall impairments in personality functioning correlated more strongly with overall interpersonal problems than clinician‐observed impairments, possibly because of common method variance. Regarding interpersonal functioning, there were no significant differences between the self‐report and the external report regarding amplitude or angular shift, indicated by the overlap of the CIs of both parameters for both measures (see Table 1). Self‐functioning could not be assessed due to unacceptable *R* ^2^. Moreover, applying the SSM in a large, naturalistic inpatient sample with high PD prevalence enabled a fine‐grained analysis of how AMPD constructs map onto interpersonal problems. Importantly, compared to published age‐ and gender‐specific normative data (Horowitz et al. 2016), mean scores on most IIP‐C octant scales in the present sample were elevated and, in some cases, substantially so. PA was the only scale to fall below the normative mean. These deviations highlight the clinical severity and interpersonal difficulties characteristic of treatment‐seeking individuals, thereby strengthening the interpretability and external validity of our findings.

## 6 Conclusion

In conclusion, both AMPD Criteria A and B were associated with general interpersonal distress. However, Criterion A domains did not map cleanly onto the IPC's meta‐concepts of agency and communion; only a subset yielded interpretable circumplex profiles, primarily in the interpersonal functioning domain. Criterion B domains generally showed clearer and more specific associations, with most mapping onto domineering and cold interpersonal problems but showing little association with nonassertive or maladaptive warm problems.

These results support and extend previous evidence of empirical links between the AMPD and the IPC, highlighting the IIP‐C's value for comprehensive assessment of interpersonal problems in PD, and, given ICD‐11's similar dimensional approach, suggest applicability beyond the AMPD. Incorporating the IIP‐C may enhance the assessment of interpersonal problems and help identify individuals at risk of personality pathology. Further research should clarify AMPD–IPC overlaps and distinctions to ensure that all clinically relevant interpersonal problems are addressed in treatment. In particular, future studies should examine which dimensions of interpersonal problems are most clinically relevant and predictive for treatment planning and long‐term outcomes.

## Author Contributions

P.L.A.S.: conceptualization, data curation, formal analysis, investigation, methodology, writing – original draft, writing – review and editing. J.I.K. and M.A.R.: conceptualization, data curation, investigation, methodology, writing – original draft, writing – review and editing. S.N. and J.W.: writing – review and editing. S.G., A.J., and F.P.: conceptualization, writing – review and editing.

## Ethics Statement

The data for this research article were derived as part of a naturalistic study (German Clinical Trial Register ID: DRKS00019821) and approved by the local ethics committee of the Faculty of Medicine, LMU Munich (EK‐No. 713‐15). Before the start of the study, all participants gave written informed consent.

## Conflicts of Interest

F.P. is a member of the European Scientific Advisory Board of Brainsway Inc., Jerusalem, Israel, and the International Scientific Advisory Board of Sooma, Helsinki, Finland. He has received speaker's honoraria from Mag&More GmbH and the neuroCare Group. His laboratory has received support with equipment from neuroConn GmbH, Ilmenau, Germany, and Mag&More GmbH and Brainsway Inc., Jerusalem, Israel. All other authors declare no conflicts of interest.

## Supporting information


**Table S1:** Zero‐order correlations between AMPD Criterion A/B scales and IIP‐C octant scores.

## Data Availability

The data supporting the findings of this study are available from the corresponding author upon reasonable request.
