# Supplementary material for: Interpersonal Problems and the Alternative Model of Personality Disorders: An Investigation Using the Interpersonal Circumplex
Source: Personal Ment Health. 2025 Nov 19;19(4):e70045. doi: 10.1002/pmh.70045 (PMC12629892; doi:10.1002/pmh.70045)
Supplement: Supplementary file 1 — Table S1: Zero‐order correlations between AMPD Criterion A/B scales and IIP‐C octant scores. [file PMH-19-0-s001.docx]

Supplement Table S1

*Zero-order correlations between AMPD criterion A/B scales and IIP-C octant scores*

| Scale | PA | BC | DE | FG | HI | JK | LM | NO |
| --- | --- | --- | --- | --- | --- | --- | --- | --- |
| STiP-5.1 total score | 0.24** | 0.33*** | 0.33*** | 0.33*** | 0.21* | 0.15 | 0.27** | 0.25** |
| Self-functioning | 0.13 | 0.26** | 0.28** | 0.37*** | 0.27** | 0.22* | 0.26** | 0.16 |
| Identity | 0.15 | 0.23** | 0.12 | 0.30*** | 0.25** | 0.24** | 0.36*** | 0.22* |
| Self-direction | 0.17 | 0.22** | 0.34*** | 0.34*** | 0.21* | 0.16 | 0.13 | 0.05 |
| Interpersonal functioning | 0.32*** | 0.34*** | 0.31*** | 0.21* | 0.09 | 0.06 | 0.20* | 0.30*** |
| Empathy | 0.40*** | 0.34*** | 0.19* | 0.07 | –0.04 | -0.05 | 0.13 | 0.33*** |
| Intimacy | 0.20* | 0.28** | 0.36*** | 0.26** | 0.16 | 0.14 | 0.21* | 0.17 |
| LPFS-BF 2.0 total score | 0.51*** | 0.53*** | 0.53*** | 0.44*** | 0.34*** | 0.33*** | 0.46*** | 0.45*** |
| Self-functioning | 0.33*** | 0.36*** | 0.38*** | 0.39*** | 0.36*** | 0.39*** | 0.49*** | 0.34*** |
| Interpersonal functioning | 0.52*** | 0.52*** | 0.50*** | 0.35*** | 0.21** | 0.18* | 0.29*** | 0.41*** |
| PID-5-BF+ total score | 0.55*** | 0.52*** | 0.42*** | 0.34*** | 0.22** | 0.26** | 0.48*** | 0.52*** |
| Negative affectivity | 0.36*** | 0.34*** | 0.14 | 0.21** | 0.25** | 0.27*** | 0.52*** | 0.59*** |
| Detachment | 0.09 | 0.36*** | 0.66*** | 0.58*** | 0.31*** | 0.28*** | 0.22** | –0.12 |
| Antagonism | 0.44*** | 0.30*** | 0.05 | –0.02 | –0.10 | –0.04 | 0.07 | 0.39*** |
| Disinhibition | 0.44*** | 0.37*** | 0.25** | 0.15 | 0.10 | 0.22** | 0.33*** | 0.44*** |
| Psychoticism | 0.44*** | 0.31*** | 0.22** | 0.15 | 0.03 | 0.08 | 0.27*** | 0.33*** |
| *Note*. Pearson zero-order correlations (*r*); significance: **p* < .05, ***p* < .01, ****p* < .001. IIP-C octants: PA = Domineering, BC = Vindictive, DE = Cold, FG = Socially avoidant, HI = Nonassertive, JK = Exploitable, LM = Overly nurturant, NO = Intrusive. Criterion A: STiP-5.1 (clinician) and LPFS-BF 2.0 (self-report). Criterion B: PID-5-BF+. Pairwise complete observations; median *N* = 151. | | | | | | | | |
